# Rosace-AA: enhancing interpretation of deep mutational scanning data with amino acid substitution and position-specific insights

**DOI:** 10.1093/bioadv/vbaf218

**Published:** 2025-09-17

**Authors:** Jingyou Rao, Mingsen Wang, Matthew K Howard, Christian Macdonald, James S Fraser, Willow Coyote-Maestas, Harold Pimentel

**Affiliations:** Department of Computer Science, UCLA, Los Angeles, CA 90095, United States; Department of Mathematics, Baruch College CUNY, New York, NY 10010, United States; Department of Bioengineering and Therapeutic Sciences, UCSF, San Francisco, CA 94143, United States; Tetrad Graduate Program, UCSF, San Francisco, CA 94143, United States; Department of Pharmaceutical Chemistry, UCSF, San Francisco, CA 94158, United States; Department of Bioengineering and Therapeutic Sciences, UCSF, San Francisco, CA 94143, United States; Department of Bioengineering and Therapeutic Sciences, UCSF, San Francisco, CA 94143, United States; Department of Pharmaceutical Chemistry, UCSF, San Francisco, CA 94158, United States; Quantitative Biosciences Institute, UCSF, San Francisco, CA 94158, United States; Department of Bioengineering and Therapeutic Sciences, UCSF, San Francisco, CA 94143, United States; Quantitative Biosciences Institute, UCSF, San Francisco, CA 94158, United States; Department of Computer Science, UCLA, Los Angeles, CA 90095, United States; Department of Computational Medicine, David Geffen School of Medicine, UCLA, Los Angeles, CA 90095, United States; Department of Human Genetics, David Geffen School of Medicine, UCLA, Los Angeles, CA 90095, United States

## Abstract

**Motivation:**

Proteins are dynamic systems whose function and behavior are sensitive to environmental conditions and often involve multiple cellular roles. Deep mutational scanning (DMS) experiments generate extensive datasets to capture the functional consequences of mutations. However, the sheer volume of data presents challenges in visualization and interpretation. Current approaches often rely on heatmaps, but these methods fail to capture the nuanced effects of amino acid substitutions, which are essential for understanding mutational impact.

**Results:**

To address this, we extend the Rosace framework with Rosace-AA, a model that incorporates both position-specific information and amino acid substitution trends. Using substitution matrices like BLOSUM90, Rosace-AA infers an interpretable score from the raw counts of growth-based DMS data, on both protein-level and at the position-level while simultaneously inferring the effect of each variant. We demonstrate its utility across datasets, including OCT1 and MET kinase, showing that Rosace-AA highlights key positions where mutations deviate from expected substitution patterns and captures functionally relevant variation in protein behavior across multiple DMS screens. These results suggest that Rosace-AA enables more robust and interpretable analysis of complex DMS datasets.

**Availability and implementation:**

An implementation of Rosace-AA as an R package and vignettes can be found at this repository: https://github.com/pimentellab/rosace-aa. Scripts for processing data and generating figures in this article are also available on GitHub (https://github.com/roserao/rosaceaa-paper-script).

## 1 Introduction

In protein biology, a central question is whether a variant’s position within a protein or the type of amino acid (AA) substitution has a greater impact on function (Fowler and Fields 2014). Research has shown that a variant’s position and substitution type can affect the mutation outcome.

Studies have demonstrated the importance of position, as certain amino acids play key roles in maintaining structural integrity, stability, or mediating interactions (Weile *et al.* 2021, Coyote-Maestas *et al.* 2022, Gersing *et al.* 2023, Estevam *et al.* 2024). For example, in G-protein-coupled receptors (GPCRs), some mutations can severely impair function due to their critical roles in ligand recognition and signal transduction (Jones *et al.* 2020, Howard *et al.* 2024). In contrast, mutations in surface-exposed loops are generally less consequential, as these regions are often peripheral to core functionality (Jones *et al.* 2020, Howard *et al.* 2024). Other investigations highlight that the nature of the substitution itself is equally critical (Dunham and Beltrao 2021, Munro and Singh 2021). Properties such as charge, hydrophobicity, size, and hydrogen-bonding potential could influence the functional impact of a mutation. Swapping amino acids with markedly different properties, such as changing a hydrophobic residue to a polar one, can disrupt function, especially if the original residue supports the protein’s core structure or active site (Wilbur 1985, Henikoff and Henikoff 1992). On the other hand, substitutions that maintain similar characteristics are often better tolerated.

Importantly, the interaction between the effects of position and substitution type also affects the outcome. A substitution’s impact often depends on its location within the protein structure: a change in a conserved and functionally critical region may lead to significant disruption, whereas the same substitution in a less crucial area might be more tolerable. This suggests that both factors matter, and that quantifying their respective impacts could provide a clearer understanding of mutation effects and relationships between position and substitution type.

Researchers have developed simple statistics to explore these questions. For example, using deep mutational scanning (DMS) experiments to simultaneously measure the effect of hundreds of thousands of variants and calculating the average effect of mutations at each position or counting functionally significant variants offers insights into functional hotspots and sensitivity (Macdonald *et al.* 2023, Estevam *et al.* 2024, Yee *et al.* 2024). Others have examined amino acid substitution trends across positions, drawing on substitution matrices such as BLOSUM (Henikoff and Henikoff 1992) and PAM (Wilbur 1985). From the perspective of multiple sequence alignment of protein sequences, methods such as direct coupling analysis (Weigt *et al.* 2009) and its many variants are developed to disentangle the direct or indirect correlations between positions. Most recent efforts include applying deep learning methods to predict the pathogenicity of a variant, notably AlphaMissense (Cheng *et al.* 2023). Those tools have varying degrees of success in mutation effect prediction, and their validity is backed up by experimental data. It is thus essential to have a tool that processes raw data from such experiments (e.g. DMS) to produce reliable empirical estimates of mutation effects. Our tool is the first statistical inference tool that learns the interaction between position and amino acid substitution while conducting statistical inference from raw DMS count data.

We extend the Rosace framework (Rao *et al.* 2024) to create a holistic framework which provides an intuitive interpretation of the interplay between position and substitution variant effects using growth-based DMS data. Our approach incorporates both position-specific information and amino acid substitution trends by decomposing variance into three components: (i) average effect at a position, (ii) contribution of amino acid substitution, and (iii) the remaining unexplained variation. This extended framework, termed Rosace-AA, provides three summary statistics for each position: the position-specific score, the position-specific scaling factor (susceptibility) for amino acid substitution effects, and the residual variance not explained by position or amino acid substitution. Those statistics quantify the effects of position, substitution, and their interaction, facilitating comparison within a protein and among experiments of the same protein under different conditions. For simplicity, we group amino acid substitutions based on BLOSUM90 in this article, but the framework is flexible and can accommodate any user-defined substitution grouping.

Using Rosace-AA, we show that different protein domains exhibit distinct decomposition structures, as reflected by the relation of summary statistics across positions. We demonstrate such utility by applying Rosace-AA to an OCT1 DMS (Yee *et al.* 2024) to identify positions where variance deviates from global amino acid substitution expectations, emphasizing positions that disrupt local interactions and exhibit specific amino acid effects. Protein residues with high unexplained variance also suggest difficulty in prediction. Applying Rosace-AA to the MET dataset (Estevam *et al.* 2024), a multiscreen analysis across 11 inhibitors and dimethyl sulfoxide (DMSO, the control cell environment), it effectively summarizes multiphenotype screens by identifying positions with distinct sensitivities or resistance profiles across various inhibitors. Unlike the OCT1 dataset, which focuses on differentiating positions based on alignment with global amino acid substitution expectations, the MET dataset application highlights Rosace-AA’s strength in capturing diverse phenotypic responses across multiple conditions, revealing functional hotspots with specific inhibitor interactions.

## 2 Results

### 2.1 Rosace-AA models both position and AA substitution effect

**Figure 1.**
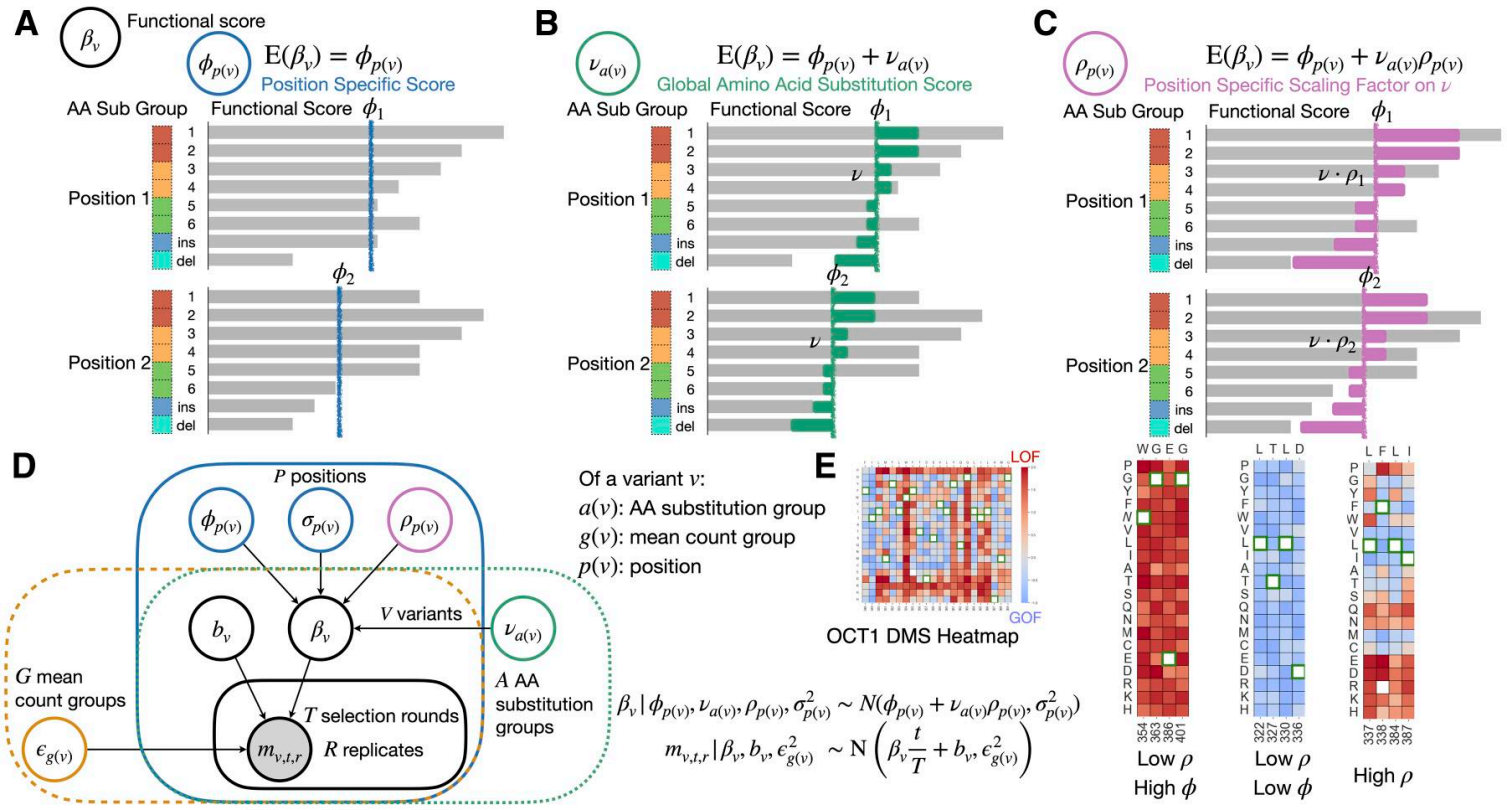
Overview of Rosace-AA model. (A–C) The functional score $\beta_{v}$ and its expectation under three models for the same variants on two positions. The labels (1—6 ins, del) indicate the mutation. The colour legend on the left indicates BLOSUM group (only four are shown; the groups are 1&2, 3&4, 5&6, and indel). The lengths of the grey bars indicate $\beta_{v}$ and the coloured bars overlaying the former bars are its expectation under the assumption of each model. (A) Model 1, original Rosace model. The prior of variant effect is the position-specific effect. (B) Model 2, Rosace with the addition of global AA effect prior. (C) Model 3, Rosace with the addition of position-scaled AA effect. (D) Plate notation of Model 3. (E) DMS functional score distribution for three types of positions, in the context of sensitivity to AA effects using OCT1 dataset as an example.

The Rosace-AA framework extends the original Rosace Bayesian hierarchical model (Rao *et al.* 2024) by modifying the prior for the variant-specific effect, denoted as $\beta_{v}$. In the original model, the prior mean of $\beta_{v}$ is represented by the position-specific effect $\phi_{p(v)}$ in Fig. 1A, where p(v) is the mapping from a variant index to its corresponding position. This position-specific prior is shared across all variants at that location, which enforces regularization and variance shrinkage (Rao *et al.* 2024), in turn, increasing sensitivity and decreasing false discovery rate.

In the extended model (Fig. 1B), an additional term, $\nu_{a(v)}$, is incorporated into the variant prior of $\beta_{v}$ to account for AA substitution effects, so that both the position and the substitution influence $\beta_{v}$. Here, a(v) denotes the mapping from the variant index to its AA substitution group. As depicted in Fig. 1B, the prior for the variant-specific score is now the sum of the position-specific score $\phi_{p(v)}$ and the global AA substitution score $\nu_{a(v)}$ (represented by the end of the green bar). Variants with lower substitution scores, such as cysteine-to-glutamic acid mutations (green group in Fig. 1B), are expected to exhibit a greater loss-of-function (LOF) effect compared to other variants at the same position. Conversely, variants with higher substitution scores, such as aspartic acid-to-glutamic acid mutations (red group in Fig. 1B), are more likely to have a neutral or gain-of-function (GOF) effect. The substitution score thus indicates the sensitivity of a function score to AA substitution.

In this study, we employ a substitution matrix to group amino acids, restricting the AA substitution analysis to single missense mutations. Insertions and deletions across positions are grouped into a pseudo-position index, with no associated AA substitution effects. Similarly, synonymous mutations, which are pre-processed and normalized to an effect of approximately zero, are assigned a constant substitution effect of zero.

While AA substitutions have a global impact (Henikoff and Henikoff 1992), their effects vary across positions, as seen in Fig. 1E for the OCT1 cytotoxicity screen (Yee *et al.* 2024). Some positions show dominant position-specific effects, while others exhibit variable substitution effects. To capture this heterogeneity, we introduce $\rho_{p(v)}$, a position-specific scaling factor (0 to 1) that modulates the influence of AA substitutions. The prior for $\beta_{v}$ combines the position-specific effect $\phi_{p(v)}$ and the scaled substitution effect $\nu_{a(v)}\rho_{p(v)}$, enabling better data fit, as illustrated in Fig. 1C.

The complete plate diagram for the third model, is shown in Fig. 1D. Notably, all parameters of the prior for $\beta_{v}$—namely $\phi_{p(v)}$, $\nu_{a(v)}$, and $\rho_{p(v)}$—are estimated jointly with $\beta_{v}$ and the rest of the model parameters. As a result, the estimation of $\beta_{v}$ is expected to have minimal bias from the experiment, and the regularization of the estimator through the prior mainly exerts on the uncertainty estimates of $\beta_{v}$. Rosace-AA is publicly available on GitHub (https://github.com/pimentellab/rosace-aa).

### 2.2 Variance decomposition of mutation effects

**Figure 2.**
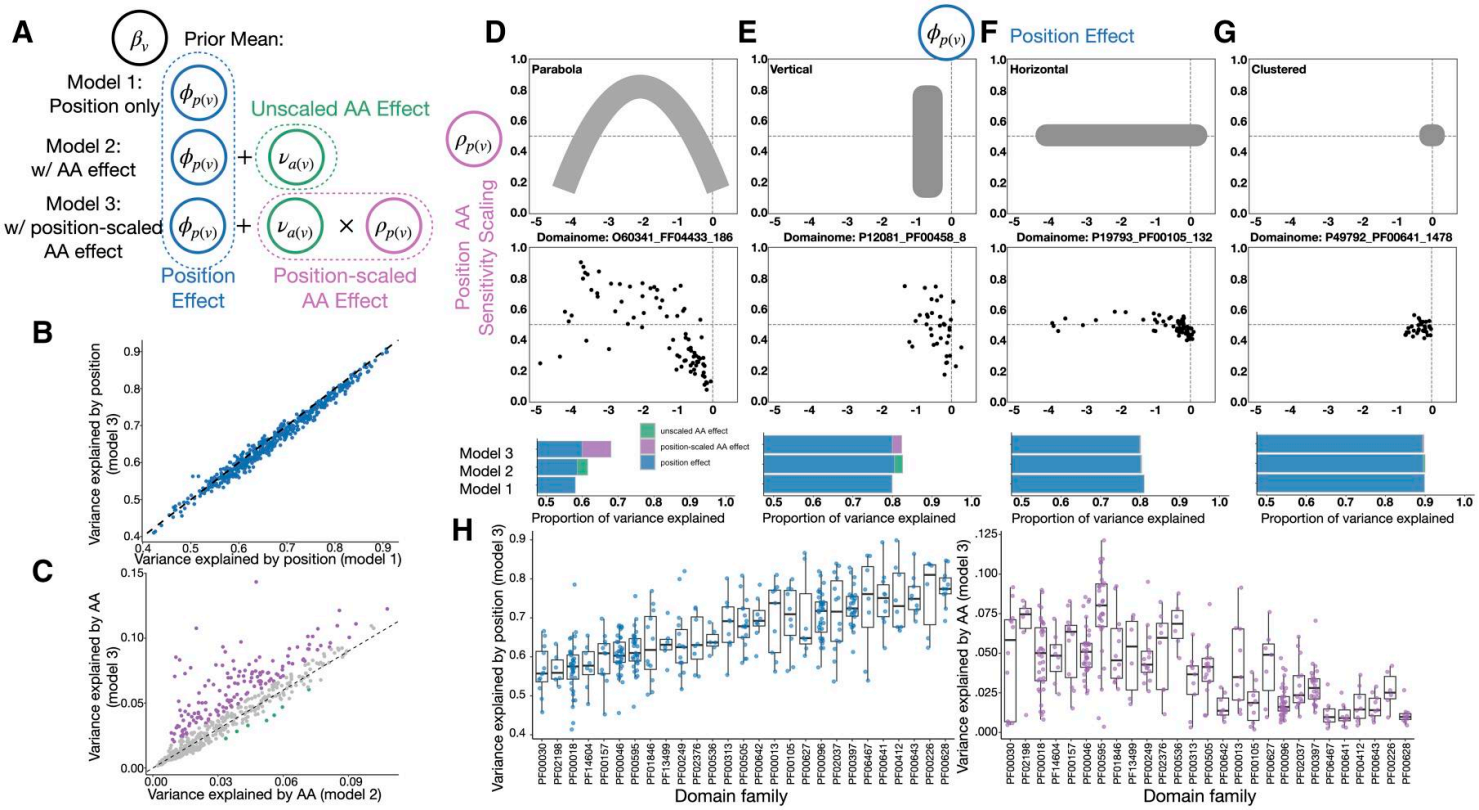
Global variance decomposition pattern and position-level effect trend applying Rosace-AA on Human Domainome 1.0 dataset. (A) Model specifications revisited. (B) Variance explained by position, comparing Models 1 and 3. (C) Variance explained by AA, comparing Models 2 and 3. Variants coloured purple (0.01 above the dotted line) have increased explained variance under Model 3 than 2. Those coloured green are the opposite (0.01 under the dotted line). Others are coloured grey. (D–G) ϕ is the position effect and ρ is the position AA sensitivity scaling coefficient. Top, the general shapes of the relation between position score and AA sensitivity scaling; Middle, an example of the relation described above; Bottom, Variance decomposition of each example domain. H, Variance explained by each factor by position, grouped by domain family. The patterns are explained in the text below.

One key novelty of the Rosace-AA method is that it achieves variance decomposition through the prior specification of a target parameter (e.g. AA substitution effects) within a Bayesian hierarchical model. This method allows characterizing how much variance in a mutation’s effect within a protein or domain (depending on the scope of the DMS experiment) is attributable to different factors, such as position, AA substitution, and other unexplained sources (Fig. 2B). Our approach extends the concept of variance decomposition, traditionally used in analysis of variance (ANOVA), by integrating it into the prior structure of a hierarchical model. Below, we explain the concept of variance decomposition in the context of ANOVA, highlight the key differences in our Bayesian formulation, and describe its application to our data.

Variance decomposition is commonly used in ANOVA. In the case of protein DMS data, we might consider the variance attributed to different positions within the protein sequence p(v) (Model 1 in Fig. 2A) and AA substitution a(v) (Models 2 and 3 in Fig. 2A).

Our model offers several advantages over ANOVA. First, while ANOVA aims to determine whether a factor significantly influences the target $\beta_{v}$ through a simple F-test, our model simultaneously performs multiple tasks. In our approach, $\beta_{v}$, the target parameter representing mutation effects, is treated as a random variable inferred from DMS count data, rather than being fixed and known *a priori*. This enables us to both estimate and perform hypothesis testing on $\beta_{v}$. Second, the factors $\phi_{p(v)}$ and $\nu_{a(v)}$ are modeled as random effects, allowing the inference of their distributions. In contrast to a typical random-effects ANOVA, where the factor is treated as random but each group within the factor is assumed identically distributed, in our model each group within a factor has its unique posterior. Further, the uncertainty in estimating $\beta_{v}$ from the DMS count data is propagated into the variance estimates for each factor.

Finally, our model allows us to compute the percentage of variance explained by each factor and to characterize the mutation effect’s variance within a protein or domain. This decomposition provides insight into how much of the observed variance is due to position, AA substitution, or unexplained local factors, which can be summarized to characterize the protein. This rich variance decomposition serves as a powerful tool for understanding the role of different factors in protein function and evolution.

### 2.3 Position effects dominate variance decomposition in human domainome data

We applied the three models from Fig. 1 to over 500 human protein domains from the Human Domainome 1.0 dataset (Beltran *et al.* 2024), which measures domain stability, a necessary condition for function. Variance explained by position effects (Model 1) and by both position and AA substitution effects (Models 2 and 3) showed that position effects dominate, explaining 40%–90% of stability variance in most proteins. This high number could be partially attributed to conservation in domains, leading to a stronger position-specific effect regardless of the substitution type compared to regions outside. Notably, variance explained by position was consistent between Models 1 and 3 (Fig. 2B), highlighting model robustness and the independence of our factor decomposition despite interdependence between position and substitution effects.

Certain domain families consistently show high variance explained by stability position effects, with PF00628 (PHD-finger), PF00226 (DnaJ domain), PF00643 (B-box zinc finger), and PF00412 (LIM domain) accounting for over 75% of the variance (Fig. 2H). In contrast, families like Beta/Gamma crystalline and PF02198 (Ets-domain) exhibit lower variance explained by position effects (below 57%), suggesting other factors contribute more to their variability.

Variance explained by global AA substitution effects (Model 2) was small, ranging from 0% to 10% (Fig. 2C). Adding the position-specific scaling factor in Model 3 increased AA substitution variance for some proteins, reaching up to 10% (annotated purple in Fig. 2C). While AA substitution effects are minor compared to position effects, their interaction with position-specific factors can be significant for certain proteins, highlighting their combined impact on stability mutation effects within protein domains.

Protein domain families with higher variance explained by position tend to have lower variance explained by amino acids, as expected. The PF00595 (PDZ domain) family, with a narrow distribution in position variance but a broad range in amino acid substitution variance, highlights this pattern. For example, while DLG4 (P78352, 60) and MPDZ (O75970, 372) both show 60% position variance, DLG4 has no significant amino acid substitution effect, whereas MPDZ explains over 12%. This difference may indicate functional specialization or structural constraints within PDZ domains, where certain proteins might be more tolerant or dependent on specific amino acid changes, reflecting nuanced aspects of protein stability.

Additionally, we tested in entire proteins (as opposed to domains), to see if the variance attributed to position effects still dominates other sources. We observe that this is indeed the case. Using datasets with raw counts from ProteinGym (Notin *et al.* 2023), the proportion of variance attributed to position effects and AA substitution effects are: 54.1%, 1.3% [CAR11_gof (Meitlis *et al.* 2020)]; 55.6%, 2.6% [CAR11_lof (Meitlis *et al.* 2020)]]; 24.3%, 0.5% [CAS9_neg (Spencer and Zhang 2017)]; 39.9%, 0.2% [CAS9_pos (Spencer and Zhang 2017)]; 31.1%, 3.1% [CD19 (Klesmith *et al.* 2019)]; 45.8%, 16.7% [RNC (Weeks and Ostermeier 2023)]; 70.5%, 1.1% [SPG1_Olson (Olson *et al.* 2014)].

### 2.4 Interaction between local position and AA substitution effect

In this section, we shift focus from the global protein-level summary statistics to an analysis of local, position-specific statistics. Conceptually, the protein or domain can be divided into four categories based on these plots: no effect, position-only, AA-substitution-only, or both. In cases where both position and AA substitution effects vary, the scatter plot typically exhibits a “downward parabola” shape (Fig. 2D). When AA substitution effects dominate but position has minimal impact, the points align vertically (Fig. 2E), suggesting uniform position effects across AA substitutions. Conversely, when AA substitution effects are neutral and the position effect varies, the points form a horizontal distribution at $\rho_{p(v)}=0.5$ (Fig. 2F). If the domain is mutation-insensitive, the points cluster near the origin (Fig. 2G).

Here, for strongly neutral or LOF positions, $\rho_{p(v)}$ is low, indicating that the position dominates regardless of the substitution. However, for mildly LOF positions, substitution plays a critical role, often depending on local structure and interactions with nearby residues, resulting in a curve that opens downward. While other shapes could theoretically emerge, these four categories capture the primary trends we observe.

These local statistics are directly produced by the Rosace-AA model. We extract two key position-level posterior distributions from the mutation effect prior. (i) The position-specific effect, $\phi_{p(v)}\in (-\infty ,\infty )$, reflects the average mutation impact at a given position, with 0 indicating neutrality and more negative values signifying a stronger loss of function (LOF). (ii) The position-specific AA substitution scaling factor, $\rho_{p(v)}\in [0,1]$, quantifies how much a position is affected by global AA substitution patterns. $\rho_{p(v)}=0$ indicates no global AA substitution effect, while $\rho_{p(v)}=1$ reflects a significant influence. Further, since the prior of $\rho_{p(v)}$ is centered at 0.5, the model defaults to 0.5 if the data is uninformative (Fig. 2F and G). This occurs either because (i) there is no global AA substitution effect $\nu_{a(v)}=0$, resulting in no overall effect, or (ii) AA substitution effects are uniform across all positions, rendering scaling unnecessary.

Figure 2D–G demonstrates the different positional effects and AA substitution sensitivity patterns we have described. Each point represents a protein position, with the x-axis showing the position-specific effect and the y-axis representing the AA sensitivity scaling factor.

We further demonstrate the global protein-level summary statistics for the four categories, specifically, the proportion of variance explained by positional and AA substitution effects. In both the “no effect” and “position only” cases, positional effects dominate, explaining over 80% of the total variance. However, in cases where AA substitution effects are significant, we observe an increase in the overall proportion of variance explained, with AA effects contributing meaningfully to the model’s fit. Notably, in Fig. 2D, the position-scaled AA effect (Model 3) provides a substantially better fit than the unscaled model (Model 2), highlighting the novelty and advantage of incorporating both positional scaled AA substitution effects in our approach. This result underscores the importance of modeling the interplay between position-specific and AA substitution effects for a more accurate representation of protein mutational landscapes.

### 2.5 Unexplained mutation effects beyond position and global AA substitution require closer examination

**Figure 3.**
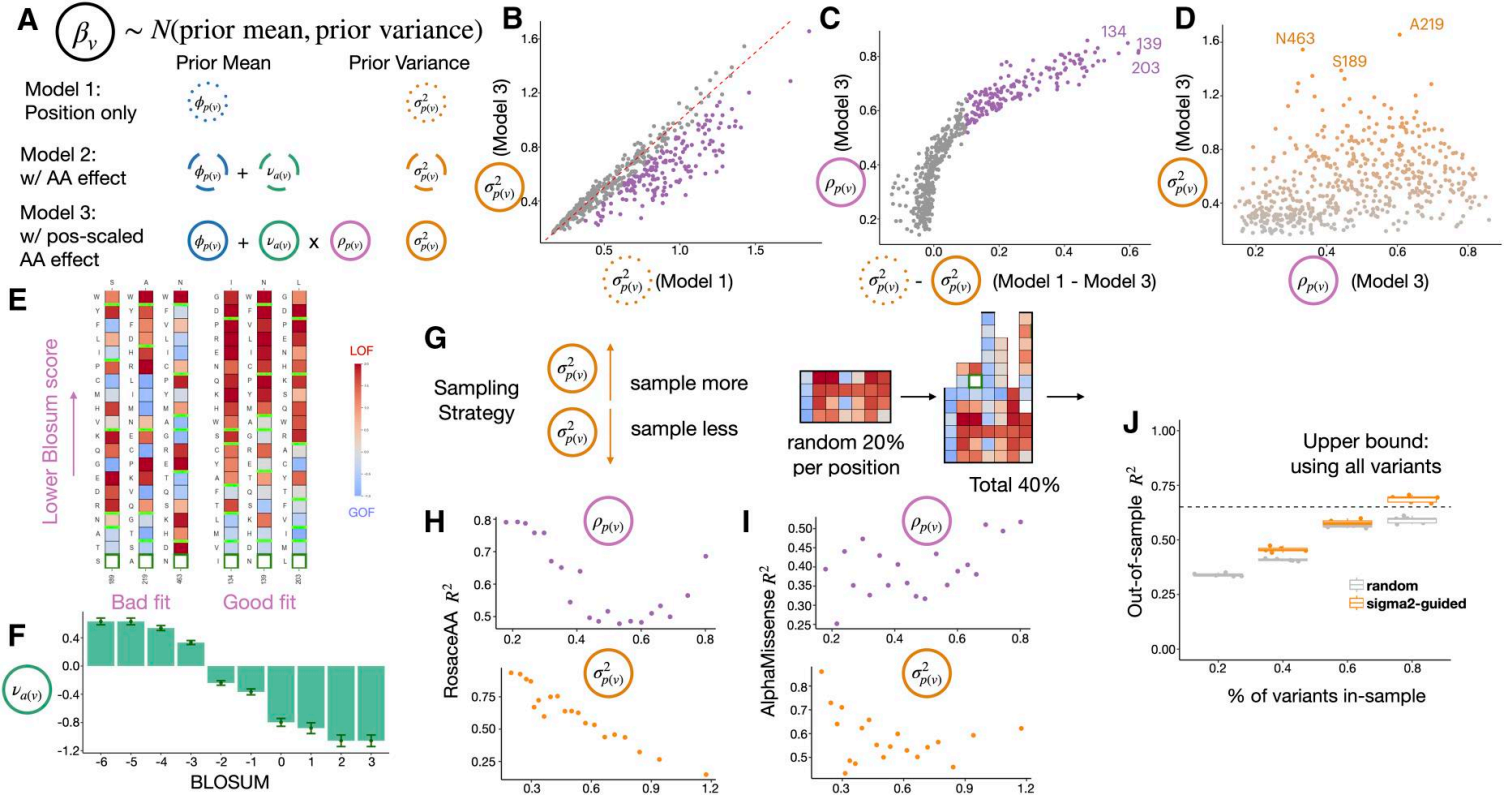
Applying Rosace-AA on OCT1 dataset. (A) Model specifications revisited. (B) Unexplained variance in Models 1 and 3. Points on the diagonal have identical σ, and purple points have significantly reduced σ in Model 3 (significantly below the diagonal). (C) Difference in unexplained variance is non-linearly attributable to AA substitution effects. Purple points (on the top-right of the plot) have significantly reduced σ in Model 3, apparently driven by larger substitution sensitivity ρ. (D) Sensitivity to AA substitution and unexplained variance by position. Orange points have high positional variance. (E) Functional score heatmap of positions with good fit or bad fit under Model 3. Variants sorted by BLOSUM score. (F) Estimated AA substitution effect by BLOSUM group. (G) Variant sampling strategy, sampling more on a position when its σ is larger. (H, I) For each ρ and $\sigma^{2}$ position ventile bins, the $R^{2}$ of β using Rosace-AA (H) and AlphaMissense (I). (J) Difference in out-of-sample *R*^2^ using random sampling or $\sigma^{2}$-guided sampling.

The Rosace-AA model captures crucial information with the Bayesian hierarchical prior of functional score $\beta_{v}$, which is modeled as a normal distribution with both prior mean and prior variance (Fig. 3A). Previously, we focused on the composition of the prior mean without leveraging the prior variance estimate $\sigma_{p(v)}^{2}$. Here, we explore the residual variance—specifically, the portion of the score that is not explained by position or global amino acid (AA) substitution—since this residual component is likely to be less predictable and may reveal important insights.

The position-specific variance $\sigma_{p(v)}^{2}$ quantifies the unexplained variance in the variance decomposition outlined in Section 2.2 of the Results. With the inclusion of variance explained by AA substitution in Model 3 (Fig. 3A), one would expect the unexplained variance to remain the same or decrease when a significant AA substitution effect is observed, indicated by a higher $\rho_{p(v)}$ value (Fig. 3B and C). This relationship is exemplified by the OCT1 cytotoxicity data (Yee *et al.* 2024) (11,432 synonymous, missense, and single-AA deletion variants).

To pinpoint the position exhibiting the greatest unexplained variance in OCT1, we plotted $\sigma_{p(v)}^{2}$ against $\rho_{p(v)}$. We expected an increase in variance explained by the global AA substitution matrix would lead to a decrease in $\sigma_{p(v)}^{2}$. Contradictory to our expectation, we observe a seemingly random pattern instead. Notably, the positions with the highest $\sigma_{p(v)}^{2}$ values (S189, A219, and N463) showed a broad range of $\rho_{p(v)}$ values (Fig. 3D). This unexpected finding prompts a closer investigation into the functional scores associated with these mutations.

To delve deeper into our analysis, We compared two groups of positions: the “poor fit” group with high $\sigma_{p(v)}^{2}$ and the “good fit” group with high $\rho_{p(v)}$ but low $\sigma_{p(v)}^{2}$. In the “good fit” group, substitutions with lower BLOSUM scores showed stronger LOF effects, aligning with expectations (Fig. 3E), while the “poor fit” group exhibited erratic LOF effects, deviating from BLOSUM trends (Fig. 3F). This discrepancy suggests that local factors, such as protein-protein interactions or conformational changes, may be influencing the functional impacts at these positions, rather than the overarching evolutionary or biophysical patterns associated with amino acid substitution. These positions are particularly compelling, as they may involve intricate local interactions within the protein or interactions with external factors, such as drugs, as seen in the OCT1 cytotoxicity screen.

### 2.6 High positional unexplained variance suggests challenges in variant effect prediction

We hypothesized that positions with the largest unexplained variance would be more difficult to predict using variant effect predictors like AlphaMissense (Cheng *et al.* 2023). This also motivates experiment designs to sample more on such positions with high unexplained variance, to compensate for the diminished efficacy of statistical methods. Conversely, suppose an experiment is constrained on the number of variants to generate and test. In that case, researchers can devote more samples to positions with larger unexplained variance and use predictors to predict other variants’ effects more reliably.

To test the hypothesis that high unexplained variance on a position indicates difficulty in variant effect prediction, we approached the prediction task as a linear regression within a Bayesian hierarchical model, using the prior mean (Model 3, Fig. 3A). This setup represents the upper bound of prediction performance for a linear regression-based method. We evaluated prediction accuracy using the *R*^2^ metric, which reflects the proportion of variance explained by position and global AA substitution (Fig. 3G). To identify which features are associated with greater prediction difficulty, we grouped variants into deciles based on position-specific AA substitution scaling factor $\rho_{p(v)}$ and variance $\sigma_{p(v)}^{2}$.

As anticipated, when grouping variants by $\rho_{p(v)}$, the *R*^2^ values display an upward parabola pattern—positions with the activation score approaching 0 or 1 have larger $R^{2}$, consistent with our model settings. However, when grouping by $\sigma_{p(v)}^{2}$, we observed that positions with higher variance were significantly harder to predict compared to those with lower variance, particularly for the highest $\sigma_{p(v)}^{2}$ decile (Fig. 3H). We also observe a statistically significant negative correlation between $\sigma_{p(v)}^{2}$ and $R^{2}$ (correlation $R^{2}$ 92%). This suggests that higher $\sigma_{p(v)}^{2}$ may indicate difficulty in variant effect prediction.

We then analysed predictions from AlphaMissense on the missense mutations (10,378 in total) of the same protein, using a continuous pathogenicity scoring scheme (0−1). Interestingly, positions with higher $\rho_{p(v)}$ values showed a slight improvement in prediction accuracy, confirming that evolutionary trends, captured by substitution matrices like BLOSUM, may enhance predictive performance. Regarding $\sigma_{p(v)}^{2}$, the lowest 10% of positions exhibited high *R*^2^ values, approaching 75%, while the highest variance positions saw this metric drop to around 30% (Fig. 3I). These findings support our initial hypothesis: positions with higher unexplained variance from DMS are indeed more difficult to predict.

Finally, we explored improving variant effect predictions when full DMS experiments are infeasible—such as for large proteins—using a sampling strategy guided by positional variance ($\sigma_{p(v)}^{2}$). The approach is simple: sample more variants from positions with high variance and fewer from those with low variance (Fig. 3J). To test this, we randomly sampled 20% of variants at each position in the first round. In the second round, we sampled another 20% either randomly or guided by $\sigma_{p(v)}^{2}$. In our implementation, we try to sample 10% more from the lowest $\sigma_{p(v)}^{2}$, 20% from the second, onto 40% from the highest. Accounting for missing observations, the latter method gained a similar number of samples as the former, which randomly samples from non-missing observations. Parameters from these datasets were used with the Rosace-AA model to predict functional scores of unsampled variants, evaluated using *R*^2^. As shown in Fig. 3J, $\sigma_{p(v)}^{2}$-guided sampling notably improved prediction accuracy over random sampling, highlighting its effectiveness.

### 2.7 Rosace-AA for multiphenotype DMS analysis

To demonstrate the utility of Rosace-AA in multiphenotype analysis, we applied the model to data from a DMS experiment on the MET kinase domain under various ATP-competitive inhibitor conditions (Estevam *et al.* 2024).

**Figure 4.**
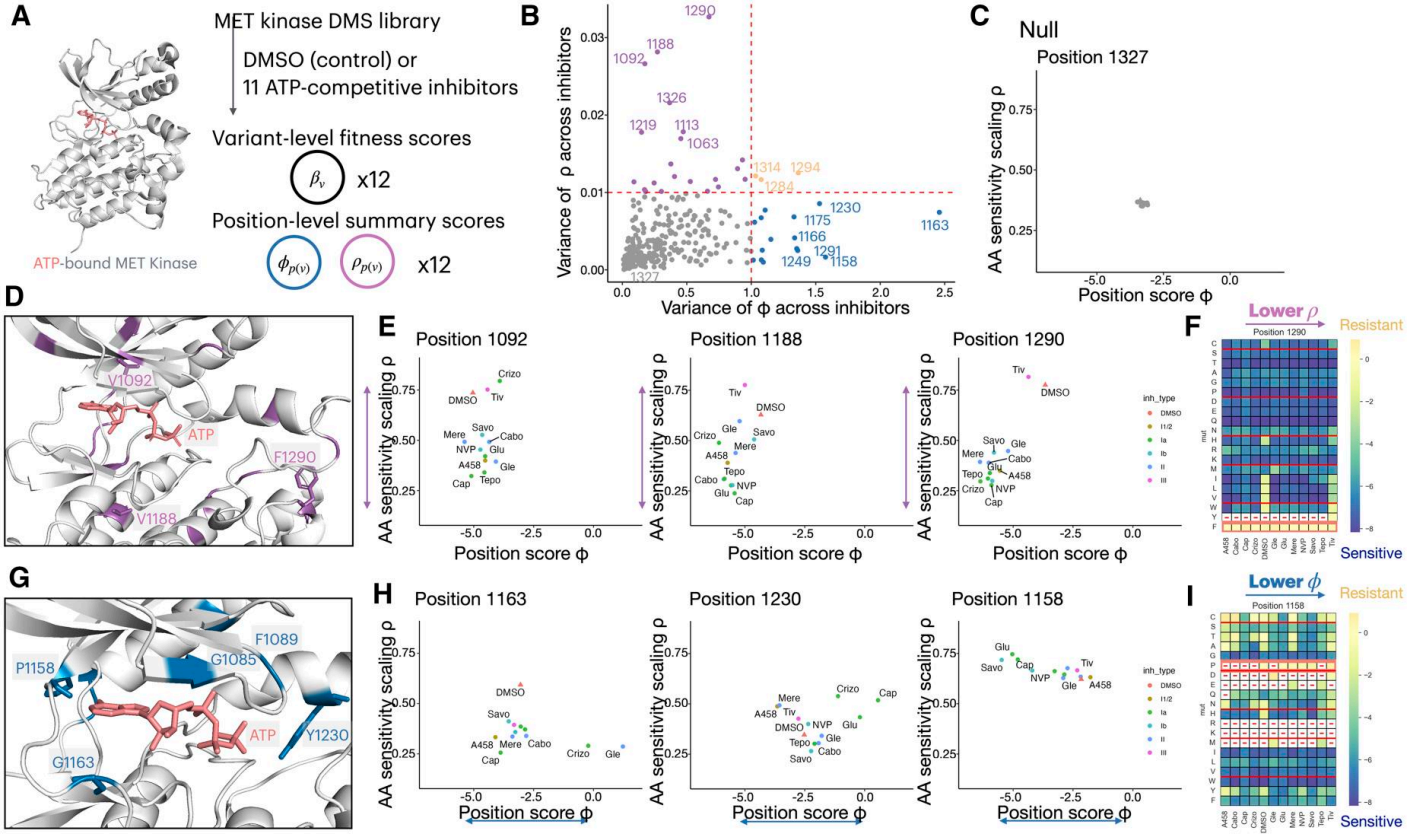
Applying Rosace-AA on MET kinase domain DMS dataset. The protein structure herein is 3DKC in PDB. In this experiment, a high fitness score implies more resistance to an inhibitor. (A) Experiment description: 12 DMS experiments are conducted on MET kinase under 12 environments, obtaining 12 sets of β, ϕ, and ρ. (B) Cross-inhibitor variance of ϕ and ρ by position, categorized according to cross-inhibitor consistency of each factor—if the variance of ϕ≥1, if the variance of ρ≥1%, or if both thresholds are reached. (C) Example of both ϕ and ρ being consistent on a position across inhibitors. (D, E) Examples of consistent ϕ but varying ρ positions. (F) Functional score heatmap of a position with consistent ϕ but varying ρ. (G, H) Examples of consistent ρ but varying ϕ positions. (I) Functional score heatmap of a position with consistent ρ but varying ϕ.

MET, a receptor tyrosine kinase (RTK) implicated in cancers like lung and gastric, drives cellular growth via ATP-dependent signaling, but mutations often confer resistance to inhibitors, requiring evaluation of multiple treatments (Estevam *et al.* 2024). In this DMS dataset, experiments were conducted under a control condition and with 11 different ATP-competitive inhibitors. For each condition, we separately computed the variant-level $\beta_{v}$ and position-level $\phi_{p(v)}$ and $\rho_{p(v)}$, using Rosace-AA (Fig. 4A).

To identify positions with inhibitor-specific effects, we computed the variance of these position-level scores and visualized them in a scatter plot. Positions with minimal variability across inhibitors represent the null condition, such as position R1327, which showed consistent behavior across all inhibitors (Fig. 4C). Positions of greater interest exhibit significant variance in either $\phi_{p(v)}$ (blue in Fig. 4B) or $\rho_{p(v)}$ (purple in Fig. 4B), indicating inhibitor-specific effects. We mapped these key positions onto the MET structure (Fig. 4D and G).

The top three positions with high variance in $\phi_{p(v)}$ were located within the ATP-binding pocket of MET (Fig. 4G), suggesting that mutations at these sites produce inhibitor-specific effects at the inhibitor-binding pocket without broadly affecting overall protein function. Each of these three positions displayed distinct resistance profiles to the different inhibitors (Fig. 4H). For example, MET inhibitors can be grouped by their binding mode to the ATP pocket and conformational preference (Estevam *et al.* 2024). Mutations to position P1158 are more sensitive to types I1/2, II, and III inhibitors and more resistant to types Ia and Ib inhibitors (this is also shown in Fig. 4I), implying that the mutations are likely blocking the binding of the latter inhibitors and facilitating that of the former ones. Position Y1230 displays a roughly opposite profile. Position G1163 is the most interesting case among the three as sensitivity to inhibitors is apparently not related to inhibitor type.

In contrast, the top three positions with high variance in $\rho_{p(v)}$ were located farther from the ATP-binding pocket, suggesting a more distal allosteric effect (Fig. 4D). Interestingly, these positions shared a common substitution profile: DMSO and Tivantinib (Tiv) inhibitors displayed high general amino acid substitution effects, while Capmatinib (Cap) showed the lowest (Fig. 4E). Further inspection of other positions with high ρ shows that Tiv generally has similar behavior to that of DMSO, in contrast to other inhibitors, probably due to its MET independence and a different inhibition mode. A detailed examination of the heatmap for position V1290 revealed that substitutions to Ile, Leu, Val, and Trp were driving these changes of $\rho_{p(v)}$ (Fig. 4F).

In summary, this MET analysis demonstrates how Rosace-AA’s position-level summary statistics can reveal nuanced insights into the functional impact of mutations across different phenotypic conditions. By capturing variance in mutation effects both locally (within critical functional sites) and distally (at allosteric positions), Rosace-AA enables the identification of mutation-specific and condition-specific functional shifts. This approach not only aids in understanding the mechanistic underpinnings of inhibitor resistance but also provides a broader framework for multiphenotype analyses, allowing researchers to explore complex genotype-phenotype relationships in various experimental contexts.

## 3 Discussion

The Rosace-AA framework provides a new powerful approach to dissecting the functional effects of mutations across diverse conditions by incorporating both position-specific information and amino acid substitution trends. Its ability to classify positions based on mutation effects and susceptibility to amino acid changes has broad applications in fields such as precision medicine, protein engineering, and drug discovery. For example, the insights gained from the MET DMS dataset highlight its potential for identifying mutation-driven resistance mechanisms in cancer therapeutics. By extending this framework to other proteins or drug-target interactions, researchers can uncover novel functional insights that inform personalized treatment strategies or guide the design of more effective inhibitors.

A current major challenge in DMS is to gain biological insight from the screen. It usually involves *ad hoc* quantitative analyses of sequence patterns and protein structure. Incorporating mutational information allows for identifying positions that statistically diverge from the background. Divergent variants and their residues are likely to have distinct roles in protein function such as binding interfaces, catalytic sites, and other crucial positions, and thus merit further inspection.

Despite its strengths, the Rosace-AA framework has limitations. First, the decomposition of variance relies on predefined substitution groupings, such as the BLOSUM90 matrix, which may not fully capture all biological contexts. The framework’s effectiveness in analyzing other protein families with distinct evolutionary or functional constraints remains to be thoroughly tested.

Future research could extend Rosace-AA by incorporating more sophisticated models of AA substitution, allowing users to input custom substitution matrices. This flexibility would enhance the framework’s applicability across diverse protein domains and evolutionary contexts, providing more biologically relevant insights. Another important direction is to model epistatic interactions with multi-mutation DMS data, enabling Rosace-AA to capture non-linear mutation effects that go beyond position-specific and substitution effects. This extension would provide a more comprehensive view of mutational impacts, especially in cases where substitution patterns are more complex. Additionally, the positional features extracted by Rosace-AA could guide more targeted subsampling of mutations, facilitating the mapping of mutational landscapes and helping prioritize regions of interest for further experimental or computational study.

## 4 Methods

### 4.1 Rosace-AA: notation and count

Following Rosace’s notation, in a growth-based DMS screen, each raw sequencing count is denoted by $c_{v,t,r}$, standing for variant, selection round, and replicate indices, respectively.

p(·) encodes the positional information of a variant. In most cases, it maps a variant to its amino acid position. There are two notable exceptions, however: (i) If information is given whether each variant is synonymous or not, all synonymous variants are grouped as a virtual “control” position. (ii) If a position has too few variants (due to missing data, for example), all variants of this position will be merged into the next position. The process is repeated if the combined position also lacks sufficient variants.

a(·) is a similar mapping that extracts the mutant corresponding to a variant, it can be substitution, insertion, or deletion. In this article, the terms “mutant” and “AA substitution” are used interchangeably.

Every possible mutant is further assigned a mutant group label, with *A* possible mutants in total. a(·) thus maps variants to such “AA substitution groups.” Since each AA substitution group contains multiple mutants, a(·) is by construction a coarser mapping than u(·).

Rosace-AA preprocesses raw sequencing counts identically as Rosace: variant filtering, missing data imputation, scale transformation, and count normalization. The resultant aligned count is denoted $m_{v,t,r}$.

### 4.2 Rosace-AA: Bayesian hierarchical model

Like Rosace, Rosace-AA assumes linear growth of the aligned count *m* with regard to time *t* (selection round):


(1) $$m_{v,t,r}|\beta_{v},b_{v},\epsilon_{g(v)}^{2}∼\text{Normal}\left(\beta_{v}t/T+b_{v},\epsilon_{g(v)}^{2}\right)$$

where the growth rate $\beta_{v}$ is treated as the functional score of the variant and $b_{v}$ as the intercept. $\epsilon_{g(v)}$ is the scale of the error, grouped by the mean group of the variant g(v) described in Rosace. The prior of each error scale is independently given by: $\epsilon_{g(v)}∼\text{InvGamma}(1,1)$.

The variant-level mean growth rate $\beta_{v}$ is modeled as:


(2) $$\beta_{v}|\phi ,\nu ,\rho ,\sigma^{2}∼\text{Normal}\left(\phi_{p(v)}+\nu_{a(v)}\rho_{p(v)},\sigma_{p(v)}^{2}\right)$$

The position-level mean effect ϕ and variance $\sigma^{2}$ are, consistent with Rosace, given weak priors: $\phi_{p(v)}∼\text{Normal}(0,1)$ and $\sigma_{p(v)}^{2}∼\text{InvGamma}(1,1)$.

The hierarchical model is solved with numeric Bayesian inference using Stan (Stan Development Team 2023). We use the default sampler offered, the No-U-Turn sampler (NUTS).

#### 4.2.1 AA-substitution-level growth rate and activation

Rosace-AA has two additional components to $\beta_{v}$: (i) AA-substitution-group-level functional effect $\nu_{a(v)}$. It can be loosely interpreted as the functional “potential” inherent to an AA substitution. This term is not directly additive with $\phi_{p(v)}$, but regulated by: (ii) position-level activation score $\rho_{p(v)}$. The AA substitution has the largest impact at positions with the highest activation scores (the most “activated”).

We use BLOSUM90 to score every possible AA substitution. A high BLOSUM score implies that the substitution is prevalent, which likely translates into less impact on protein function. Substitutions of similar BLOSUM scores are grouped so that each group covers at least 20% of positions. Insertions and deletions, not explicitly scored, are given two separate BLOSUM group labels from substitutions as their functional effects are assumed to be vastly different.

Synonymous variants are assigned the same virtual “position” and the same substitution label, whose functional effect centers to 0. Also, to avoid identification problems, we force all non-synonymous AA substitution effects ν to sum up to 0, weighted by the variant count of each AA substitution group. As opposed to equal weighting, this weighting scheme is invariant to how AA substitutions are grouped.

Recall that the AA substitution group label *A* is the “synonymous group.” Let the mean-normalized variant count of non-synonymous mutation be $w\in R^{A-1}$, then the Gaussian prior on ν is set as follows:


(3) ν−A∼Normal(0,diag(w)−1M diag(w)−1)where Mij=1i=j−1A−21i≠j, νA=0

*M* is an exchangeable but degenerate correlation matrix with rank A−2. *w* is mean-normalized so that if all non-synonymous mutation groups have the same number of variants, diag(w) is simply the identity matrix.

Activation scores $\rho_{p(v)}$ are assumed to range in [0, 1]. Intuitively, an activation score of 1 implies full activation of AA substitution effects and 0 complete inactivation. It is also assumed that most positions are moderately activated. Therefore, activation scores are given an independent and symmetric beta prior: $\rho_{p(v)}∼\text{Beta}(1.5,1.5)$

### 4.3 Data availability and processing

OCT1 (Macdonald *et al.* 2023), MET (Estevam *et al.* 2024), and the Human Domainome (Beltran *et al.* 2024) dataset are retrieved from supplementary files to their respective publications. The ProteinGym (Notin *et al.* 2023) datasets are available at https://marks.hms.harvard.edu/proteingym/ProteinGym_v1.3/DMS_ProteinGym_substitutions.zip, and only datasets with raw count information are processed. For all DMS data, variants with high-order mutation are filtered out, and only raw sequencing counts of synonymous, missense, and nonsense mutations are input into the Rosace-AA tool and processed with the default workflow: count normalization, posterior distribution sampling, and result summaries.

## Supplementary Material

vbaf218_Supplementary_Data

## Data Availability

Rosace-AA is publicly available on GitHub (https://github.com/pimentellab/rosace-aa). Scripts for processing data and generating figures are available on GitHub (https://github.com/roserao/rosaceaa-paper-script).
