# Supplementary material for: Rosace-AA: enhancing interpretation of deep mutational scanning data with amino acid substitution and position-specific insights
Source: Bioinform Adv. 2025 Sep 17;6(1):vbaf218. doi: 10.1093/bioadv/vbaf218 (PMC12809776; doi:10.1093/bioadv/vbaf218)
Supplement: vbaf218_Supplementary_Data [file vbaf218_supplementary_data.pdf]

Rosace-AA Supplementary Note

Jingyou Rao[1]

[1]Department of Computer Science, UCLA, Los Angeles, CA, USA

Methods Cont.

Rosace-AA: Bayesian Hierarchical Model

Aligned count error

The mean count of a variant is calculated by averaging the aligned counts m across time points t and replicates r. Variants are clustered based on such mean counts, the mapping describing which is denoted by $g(\cdot)$. The clustering is inspired by the idea that a low mean count is more susceptible to error during sampling, but Rosace-AA makes a weaker assumption that variants with similar mean counts have similar error scales ϵ in Equation **??**. The prior of each error scale is independently given by: $\epsilon_{g(v)} \sim \text{InvGamma}(1, 1)$

$$\epsilon_{g(v)} \sim \text{InvGamma}(1, 1) \tag{1}$$

Numeric Bayesian inference

The hierarchical model described above is not immediately analytically tractable. Instead, we opt to conduct numeric Bayesian inference using Stan [**?**]. We use the default sampler offered, the No-U-Turn sampler (NUTS), which is a variant of the Hamiltonian Monte Carlo (HMC) algorithm. HMC reduces the correlation between successive samples compared to other Monte Carlo methods such as the Metropolis-Hastings Monte Carlo (MCMC) algorithm, resulting in more efficient sampling of the parameter space and thus reduced computation time for a given level of error.

Through sampling from the posterior distribution, many quantities of interest can be obtained. In the context of this paper, the functional score β_v of each variant can be estimated (*maximum a posteriori*) and the user may be interested in identifying variants with functional scores the most divergent from that of the wild-type.

Rosette-AA: Family of Simulation Frameworks

In the Rosace paper, we introduced a data-driven growth-based DMS data simulation framework, Rosette, to complement real data in benchmarking the performance of Rosace-AA and other growth-based DMS data analysis tools. Following the pattern, we have developed a family of simulation frameworks with different sets of underlying assumptions, colloquially named Rosette-AA, to test the robustness of our models.

While Rosette and Rosette-AA have varied assumptions on how data is generated, both accept parameters that are inferred from real data. The simulation generates synthetic scores in different manners from the model, thus it serves as a robust test for the performance of models.

To avoid an unfair comparison, we designed several data simulation frameworks, based on several sets of assumptions that are increasingly complex. All frameworks begin with applying DMS analysis tools (e.g. Rosace, Rosace-AA, or OLS) to experimental data to find the functional score $\hat{\beta}_v$ of each variant v. The latent score κ_v of a variant is obtained by applying a scenario-specific transformation on all variants' functional scores. Variants are then ranked on their latent score (ties being broken randomly), labeled as negative, neutral, or positive according to their quantile, and assigned a score based on their functional identity. Here, positive and negative refer to the numeric sign - whether a negative variant is gain-of-function or loss-of-function depends on the experiment design.

The scenario-specific latent score κ_v reflects the underlying assumptions of a scenario. The scenarios, ordered by ascending complexity of their assumptions, are as follows:

$$\kappa_v = \hat{\beta}_{RP(v)} \qquad \text{(Scenario 1: all random)}$$

where $RP(v)$ is a random permutation of variant indices. In this simplest scenario, the scenario-specific score is the randomly shuffled functional score, so that functional identities (negative, neutral, or positive) are effectively assigned randomly.

$$\kappa_v = \frac{1}{\|p^{-1}(p(v))\|} \sum_{v' \in p^{-1}(p(v))} \hat{\beta}_{v'} \qquad \text{(Scenario 2: position-only)}$$

Scenario 2 assumes the effect of a variant only depends on the positional effect. Therefore, its latent score is the average of functional scores in the same position.

The last three scenarios have similar structures:

$$\begin{aligned}\kappa_v =& \frac{1}{\|p^{-1}(p(v))\|} \sum_{v' \in p^{-1}(p(v))} \hat{\beta}_{v'} \\ &+ \frac{1}{\|b^{-1}(b(v))\|} \sum_{v' \in b^{-1}(b(v))} \hat{\beta}_{v'} \cdot a(p(v))\end{aligned} \qquad \text{(Scenarios 3-5: position and substitution)}$$

Scenarios 3 to 5 add to the latent score the average functional scores over variants with the same amino acid substitution, with a position-level regularization term $a(p(v)) \in [0, 1]$ (i.e. "activation"). All three scenarios assume that amino acid substitution plays a role in determining the outcome.

Scenario 3 assumes $a(w) = 1$ so that there is no regularization on the mean substitution effect. It assumes that mutation effects are invariant across positions. In other words, mutation effects are assumed to be global.

Scenario 4 assumes $a(w) = \text{Bernoulli}(0.5)$, allowing each position to be differently affected by mean substitution effects while assuming no further structure on the activation.

Scenario 5 assumes a deterministic activation on each position with $a(w) = \mathbb{1}_{\hat{\phi}_w > \phi_{0.75}} + \mathbb{1}_{\hat{\phi}_w \leq \phi_{0.25}}$. It assumes positions with very high or very low position-mean functional scores are activated differently from those with more average position-mean functional scores. Note that this formulation is statistically indistinguishable from $a(w) = \mathbb{1}_{\hat{\phi}_w \leq \phi_{0.75}} \cdot \mathbb{1}_{\hat{\phi}_w > \phi_{0.25}}$.

Using the posterior distribution of each variant's estimated functional score $\hat{\beta}_v$, we use Bayesian hypothesis testing with its local false sign rate (LFSR) [?] to assign the estimated functional identity. In case the proportion of positive (negative) variants is too low, all positive (negative) variants will be merged into the neutral group. We have tested proteins in which all three groups can be identified, only neutral and positive groups are identified (e.g. OCT1 drug cytotoxicity screen[?]), only neutral and negative groups are identified (e.g. MET kinase domain DMS with IL3[?]), and all variants are classified as neutral (e.g. MET kinase domain DMS without IL3). We assume the variants' functional scores of each functional identity group are Gaussian, whose parameters can be estimated using the estimated functional scores of variants from that group. Variants are then ranked on their scenario-specific latent scores κ_v. Variants with high latent scores $\hat{\beta}_v$ are assigned a simulated functional score $\tilde{\beta}_v$ drawn from the positive distribution (if the positive functional identity group exists). The opposite applies to variants with low latent scores (if the negative functional identity group exists). The remaining variants' simulated scores are drawn from the neutral distribution. Note that a variant with a high estimated functional score $\hat{\beta}_v$ does not necessarily draw its simulated functional score $\tilde{\beta}_v$ from the positive distribution, as its latent score κ_v can be low.

Once the simulated functional scores $\tilde{\beta}_v$ are generated for each variant, we follow the same process in `Rosette` to generate the simulated raw count $\tilde{m}_v$.

Simulation Results

Here, we present the simulation results with summary statistics from the OCT1 dataset.

We first confirm that the `Rosace-AA` model achieves comparable inference performance to `Rosace` in terms of false discovery rate and sensitivity (Supp. Fig. 1A). Next, we examine the position and amino acid

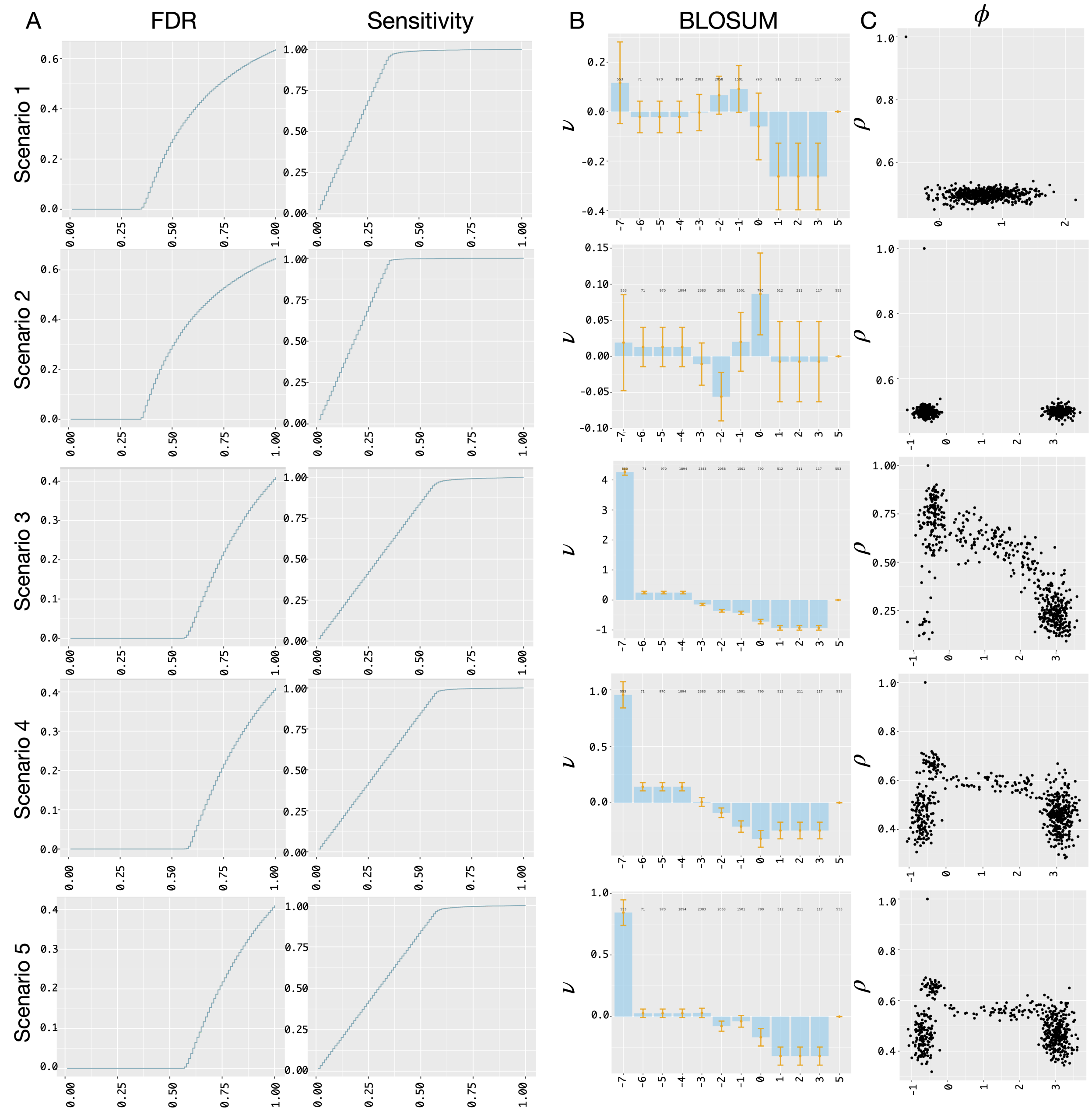

Figure 1: Simulation results with `Rosace-AA`. A: False discover rate (FDR) and sensitivity using `Rosace` (red) and `Rosace-AA` (blue) under the five simulations, each generated by a "scenario" described in the main text, are identical. The two lines overlap. B: Global amino acid substitution effects (ν) learned under different scenarios. Error bars indicate the standard error of our estimate of ν. C: The scatterplot of estimated ϕ and ρ. Each position is represented with a dot. The general shape of each scatterplot reflect the underlying assumptions of each scenario.

substitution effect trends learned by the model. As expected, the global amino acid substitution effects are close to zero for scenarios 1 and 2, but markedly significant for scenarios 3, 4, and 5 (Supp. Fig. 1B). The scatterplots of position effects versus position-level amino acid sensitivity reveal clear differences across the five scenarios. In scenarios 1 and 2, no amino acid sensitivity is detected; however, scenario 2 exhibits a clear separation of position effects (neutral vs. gain-of-function), while scenario 1 remains uniformly distributed.

In scenarios 3–5, we observe a parabolic pattern, with scenarios 4 and 5 displaying cleaner clusters of low AA sensitivity in extreme GOF and LOF regions (Supp. Fig. 1C).

References
